# Development and validation of a rapid and easy-to-perform point-of-care lateral flow immunoassay (LFIA) for the detection of SARS-CoV-2 spike protein

**DOI:** 10.3389/fimmu.2023.1111644

**Published:** 2023-02-23

**Authors:** Shamim Mohammad, Yuxia Wang, John Cordero, Christopher Watson, Robert Molestina, Sujatha Rashid, Rebecca Bradford

**Affiliations:** American Type Culture Collection, Manassas, VA, United States

**Keywords:** lateral flow immunoassay, rapid test, point-of-care testing, SARS-Cov-2 spike protein, monoclonal antibodies

## Abstract

Development and validation of rapid and easy-to-perform diagnostics continue to be a high priority during the current COVID-19 pandemic. Although vaccines are now widely available, early detection and consistent transmission control provide ideal means to mitigate the spread of SARS-CoV-2. Nucleic acid-based real‐time PCR tests are widely acknowledged as the gold standard for reliable diagnosis of COVID-19 infection. These tests are based on detecting viable or nonviable viral nucleic acids. SARS-CoV-2 spike protein is an alternative and ideal target for SARS-CoV-2 diagnosis in the early phase of infection, but point-of-care kits to detect the SARS-CoV-2 spike protein are limited. Here we describe a rapid and convenient method based on Lateral Flow Immunoassay (LFIA) to detect SARS-CoV-2 spike proteins, including SARS-CoV-2 variants (A.23.1, B.1.1.1, 1.617.2, B.1.1.7, B.1.351, P.1, N501Y, R.1, P681H, P3, UK, and South African) within 5 to 10 minutes. We generated highly specific monoclonal antibodies (mAbs) against rationally designed SARS-CoV-2 spike protein. Matched pair mAbs were selected by epitope mapping and employed as antigen capture reagents by spotting onto a nitrocellulose membrane and as detector reagents by conjugation with colloidal gold nanoparticles. We evaluated the performance of the LFIA using recombinant spike proteins of SARS-CoV-2 and several SARS-CoV-2 variants. The specificity of the LFIA was assessed using heat-inactivated SARS-CoV-2 and related human coronaviruses (HCoV-OC43, HCoV-229E, HCoV-HKU1, and HCoV-NL63) and an FDA-approved respiratory pathogens (RP) panel. The assay exhibited 98% specificity and acceptable performance with respect to the minimum limit of detection (25 ng/test) in validation tests. This new LFIA provides improved performance for the early diagnosis of SARS-CoV-2, particularly for home monitoring and in situations with limited access to molecular methods.

## Introduction

Coronavirus disease 2019 (COVID-19) is caused by severe acute respiratory syndrome-related coronavirus 2 (SARS-CoV-2). COVID-19 spread over 223 countries and was declared a pandemic on March 11, 2020, by the World Health Organization (1). Diagnosis of SARS-CoV-2 is of epidemiological interest and is relevant for prognosis in individual patients (2). There are two broad categories of SARS–CoV-2 tests: those that detect the virus itself and those that detect the host response to the virus (3, 4). The incubation period of COVID-19 varies with age. The middle-aged population (41 to 60 years) has the shortest incubation period compared to other populations, particularly the elderly population (≥ 61 years) and those aged 18 to 40 years. The median incubation time is estimated to be 6.0 days globally, with symptoms expected to be present within 12 days of infection. Symptoms of COVID-19 are similar to other viral respiratory diseases and include fever, cough, and shortness of breath. Variant mutations may affect the incubation period of COVID-19. For example, the incubation period for lineage B.1.617.2 is shortened to an average of 4.4 days (5). The antigens are generally detectable in upper respiratory specimens during the acute phase of infection. Positive results indicate the presence of viral antigens, but clinical correlation with patient history and other diagnostic information is necessary to determine infection status. Positive results do not rule out bacterial infection or co-infection with other viruses.

Antigenic tests are the only tools developed as “at-home” tests available without a prescription or requiring assistance from a specialist (6). Research on antigen detection tests for COVID-19 is ongoing but with limited results. Immunodiagnostic approaches are often hampered by the lack of specificity linked to false-positive results from antigens that are well-conserved among different coronavirus species and cross-reactions with autoantibodies found in autoimmune diseases. Antigen-based tests, on the other hand, could be a nice tool for cost-effective point-of-care (POC) diagnosis in primary care settings (7, 8). Lateral flow devices are among the most established POC testing platforms successfully deployed to areas where timely medical care is challenging (9). Three lateral flow assays received emergency use authorization from the FDA, including the Becton Dickinson (BD) Veritor™ System, the Quidel Sofia 2 SARS antigen FIA and the Abbott Diagnostics BinaxNOW COVID-19 Ag Card (10, 11). All these assays function similarly by detecting the nucleocapsid (N) protein of SARS-CoV-2 from upper respiratory samples. The N-protein detection tests demonstrate a low sensitivity, which is one of the main limitations of the N-antigenic tests explaining why they may not detect all of the active coronaviruses (12, 13). However, there is a high priority on developing and validating rapid and easy-to-perform diagnostic methods to detect emerging variants that lead to similar clinical symptoms (14). Diagnostic tools based on detecting the spike protein produced by SARS-CoV-2 on the surface of the virus can provide high specificity for detecting variants.

Nucleic acid amplification tests (NAATs) are an alternative to COVID-19 immunodiagnostic tools. These detect unique viral RNA sequences in N, E, S, or RNA-dependent RNA polymerase (RdRp) genes (7, 15). Current NAATs for coronavirus, including RT-PCR, real-time reverse transcription PCR (rRT-PCR), reverse transcription loop-mediated isothermal amplification (RT-LAMP), and real-time RT-LAMP, are mainly performed by large laboratories. Still, the tests have many limitations (16–18). RT-PCR results depend heavily on the type of sample taken, with positive sampling rates varying widely between oropharyngeal swabs (32–48%), nasopharyngeal swabs (63%), bronchoalveolar lavage fluid (79–93%), sputum (72–76%) and stool (29%) (19). The tests can detect mildly symptomatic cases in the early phase of the disease, thereby identifying the most infectious individuals. However, NAATs are generally process-intensive, susceptible to contamination, and expensive. Lateral Flow Immunoassay (LFIA) antigen tests have been proposed as rapid POC diagnostics for SARS-CoV-2 infection. They have an advantage over RT-PCR assays in that they can be self-delivered by healthcare workers at home with immediate results.

Since the inception of B cell hybridoma technology in 1975, monoclonal antibodies (mAbs) have revolutionized diagnostic science due to their specificity homogeneity towards antigen epitopes and unlimited availability (20). They are also very stable and, thus, adaptable to both clinical diagnostics and sero-surveillance studies. An essential prerequisite for generating mAbs by B cell hybridoma technology is the immunization of animals, most commonly mice, with a specific target antigen. In the case of protein targets, mice immunization is traditionally accomplished using recombinantly expressed and purified proteins. Mammalian expression systems capable of providing native protein folding, and natural posttranslational modifications, have increasingly been used to reliably express proteins in their native conformation (21). SARS-CoV-2 comprises four structural proteins: spike (S), envelope (E), membrane (M), and N. The S protein is a major component of the viral surface that binds to angiotensin-converting enzyme-2 (ACE-2), the receptor for the SARS-CoV-2, enabling the virus to enter and infect cells. Therefore, the S protein is considered an excellent diagnostic target (22). However, SARS-CoV-2 expresses several mutant forms of spike protein in order to escape the immune response and achieve viral persistence (23).

Here, we describe the development and evaluation of a highly specific, sensitive, quick, and easy-to-perform LFIA to detect SARS-CoV-2 spike proteins. This assay uses colloidal gold nanoparticles to visualize the reaction and is visible within 5 to 10 minutes. A full-length, rationally designed SARS-CoV-2 glycosylated recombinant spike protein (24, 25) was used to generate mouse monoclonal antibodies (mAbs). The mAbs were then screened and characterized by enzyme-linked immunosorbent assay (ELISA), western blot, and isotyping. Three pairs of mAbs were analyzed in competitive sandwich ELISA in the presence of spike protein to identify suitable capture and detection antibody pairs (matched antibody pairs) for the development of this assay. We evaluated the assay specificity based on matched antibody pairs using heat-inactivated SARS-CoV-2 and the related human coronaviruses (HCoV) HCoV-OC43, HCoV-229E, HCoV-HKU1, and HCoV-NL63. The assay sensitivity and specificity were measured using SARS-CoV-2 full-length spike proteins and the SARS-CoV-2 variants B.1.1.7, B.1.351, P.1, B.1.526, B.1.617.2, B.1.1.1, A.23.1, N501Y, P681H, P3, R.1, and S1 protein of UK and South African variants). The cross-reactivity was assessed with FDA approved respiratory and bacterial pathogen panels. Due to the inaccessibility of clinical specimens, we adopted a “spike-and-recovery” method, using full-length spike protein and cotton swabs. The test was capable of detecting spike protein below 25 ng in the samples. We also designed the “Swab Mimicking Test” with positive and negative samples by randomly spiking recombinant spike protein into saliva swabs to mimic clinical specimens. The assay was capable of distinguishing positive and negative samples.

**Figure 1:**
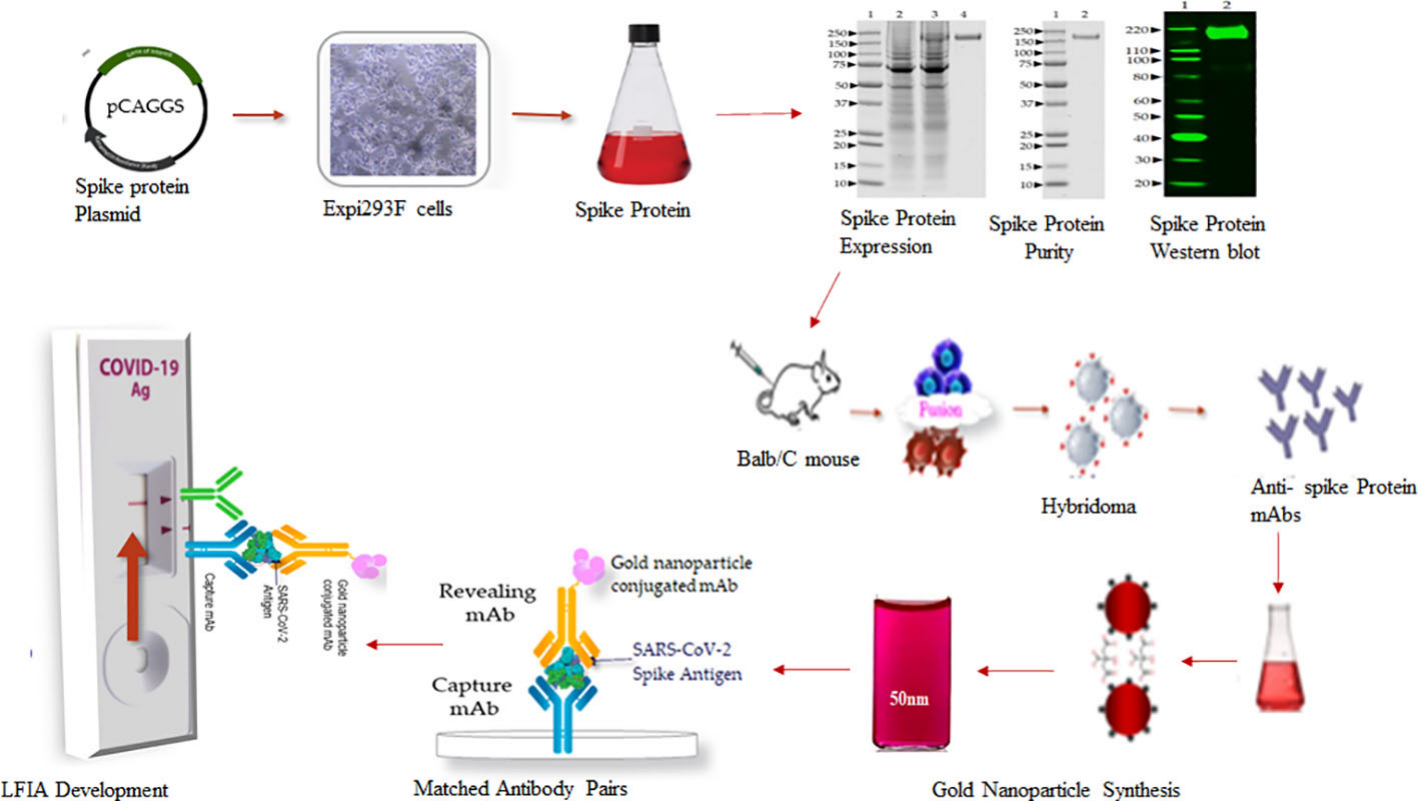
Schematic workflow for the development of SARS-Covid-2 LFIA.

A schematic workflow for the development of SARS-Covid-2 LFIA is shown in **Figure 1**.

## Materials and methods

### Ethics statement

Immunization of mice was performed in accordance with PHS Policy on Humane Care and Use of Laboratory Animals. Experiments were approved by the ATCC Institutional Animal Care and Use Committee (IACUC).

### Production and purification of recombinant full-length SARS-CoV-2 spike protein

The full-length trimeric and stabilized version of recombinant S glycoprotein protein of SARS-CoV-2 was produced by transient transfection of a mammalian expression plasmid (pCAGGS) from BEI Resources (NR-52394; www.beiresources.org). The vector for the S gene, Wuhan-Hu-1 (GenBank: MN908947), was designed for the expression of a soluble S glycoprotein (residues 1 to 1213) with a polybasic cleavage site deletion (RRAR to A; residues 682 to 685) and stabilizing mutations (K986P and V987P, wild type numbering) with a C-terminal thrombin cleavage site, T4 foldon trimerization domain, and hexa-histidine tag (26). According to the manufacturer’s recommendations, the Expi293F cells (Gibco, USA) were grown in Expi293 Expression Medium (Gibco, USA) *via* shaking in suspension using a humidified incubator at 37°C and 8% CO_2_. For the production of SARS-CoV-2 spike protein, Expi293F cells were transiently transfected using ExpiFectamine 293 transfection kit (Gibco, USA). Briefly, 1 µg/mL of the plasmid was diluted into 12 mL of Opti MEM (Gibco, USA) and 640 µL of ExpiFectamine was added in 11.2 mL Opti MEM separately, followed by mixing for 30 min at room temperature. The plasmid mix was then added onto Expi293F cells in Expi293 Expression Medium at 3 × 10^6^ cells/mL. On day 5 after transfection, the trimeric, prefusion-stabilized spike protein was harvested from the supernatant by centrifugation. The spike protein was purified on a HiTrap histidine chelating resin column (GE Healthcare) using the AKTA FPLC system (Cytiva, USA). The eluted protein was desalted with a desalting membrane (D-0655, Sigma-Aldrich) into 50 mM PBS (pH 7.4), and protein purity was confirmed by SDS-PAGE on 4-12% Bis-Tris-gel and stained with Coomassie Plus Protein Assay Reagents (Thermo Scientific). The final concentration was determined using a BCA protein estimation kit (Pierce). The endotoxin level was measured by the Limulus Amebocyte Lysate (LAL) test (Charles River Laboratories).

### Western blot analysis of purified recombinant SARS-CoV-2 spike protein

The purified SARS-CoV-2 recombinant spike protein was separated on NuPAGE Novex 4–12% Bis-Tris Gels (Invitrogen, USA) with the addition of NuPAGE Reducing Agent (Invitrogen, USA) and heating of the samples for 5 min at 95°C (reducing conditions). After electrophoresis, protein bands were transferred onto a nitrocellulose membrane, blocked with 5% non-fat dry milk in PBS containing 0.1% Tween 20, and probed with an anti-His monoclonal antibody (AF933; R & D Systems, USA), followed by Goat anti-mouse–IRDye conjugate at 1:10,000 dilution (Li-COR, USA). After the final washing steps, bands were visualized using Odyssey Infrared Imaging System (LI-COR imaging system, Biosciences, USA).

### Generation and selection of mouse monoclonal antibodies against SARS-CoV-2 spike protein

Six- to seven-week-old female Balb/c mice (Harlan Laboratories) were immunized subcutaneously with 50 µg of recombinant trimeric prefusion-stabilized spike protein emulsified with Incomplete Freund’s adjuvant (Sigma, USA) and boosted 4 weeks later with the same dose of spike protein with Complete Freund’s Adjuvant. Mice with high antibody titer as determined by ELISA were selected for fusion. Four days before the fusion, the mice received three intravenous boosts of 100 µg of spike protein without adjuvant. Mouse splenocytes were harvested and fused with Sp2/0 myeloma cells (ATCC, CRL-1581) to generate antibody-producing hybridoma cells. After hypoxanthine–aminopterin–thymidine (HAT) selective culture, the hybridomas were screened by direct ELISA using MaxiSorp microtiter plates (VWR, USA) coated with 1 µg/ml of recombinant spike protein. Five positive primary hybridoma clones were sub-cloned twice by limiting dilution to obtain stable cell lines secreting monoclonal antibodies. The class and subclass of the mAbs were identified using a mouse isotype kit (Roche, USA). Ten hybridoma cell lines exhibiting high reactivity against Spike protein (S) belonging to the IgG subclass IgG1 were selected and two of them, SpMA-01 and SpMA-02, were investigated further to develop the LFIA.

### Large-scale production, purification, and characterization of monoclonal antibodies

To produce large-scale antibodies, selected hybridomas were media-adapted in serum-free media (Gibco, USA) and introduced into a 1 L CELLine bioreactor (Corning, USA). The harvested culture supernatants from the bioreactors were concentrated using a Jumbosep™ concentrator (PALL Corporation, USA) and purified on a Protein-G column (Thermo Scientific, USA). The purified antibodies were dialyzed/concentrated on a Vivaspin column 20 concentrator (Cytiva, USA). The purities of the mAbs were analyzed using capillary gel electrophoresis (Agilent system, USA). The antibody reactivity toward the recombinant (S) was assessed using conventional ELISA and Western blot performance. A 96-well ELISA plate was coated overnight at 4°C with 50 µL of (S) diluted to 1 µg/mL in 0.05 M carbonate buffer (pH 9.6). The next day, the wells were washed with PBS and blocked with 150 µL of 5% non-fat milk protein for 1 hour at room temperature (20 - 25°C). After blocking, milk protein was removed from the plate, washed briefly with PBS, and duplicates of each mAb sample were added to test and control wells. The plates were subsequently washed with PBS-T, and bound antibodies were detected by HRP-conjugated goat anti-mouse IgG (H + L) secondary antibody (1:1000, Bethyle Lab, USA), and colorized with KPL SureBlue TMB Microwell Peroxidase Substrate (Seracare, USA). The enzyme-substrate reaction was stopped by adding 1 N sulfuric acid. Finally, the optical densities (OD) were measured at 450 nm, and the mean OD of the control wells was deducted from the mean OD of the test wells to obtain the final OD value for each sample. Western blot analysis was performed using SARS-CoV-2 full-length recombinant S, HCOV-HKU1, Middle East respiratory syndrome (MERS) coronavirus, SARS-Related Coronavirus-2, SARS-CoV-2 S receptor binding domain (RBD) recombinant protein, and Vero-Cell E6 Lysate as a negative control. Samples were separated on NuPAGE Novex 4–12% Bis-Tris Gels (Invitrogen, USA) under reducing conditions with the addition of NuPAGE Reducing Agent (Invitrogen, USA) and heating of the samples for 5 min at 95 C. After electrophoresis, protein bands were transferred onto nitrocellulose membranes. The membrane was blocked with 5% non-fat milk protein and probed with selected anti-S mAbs followed by an IRDye conjugated goat anti-mouse IgG (H + L) secondary antibody (LICOR, USA). The specific bands were visualized using the LICOR Odyssey system.

### Epitope mapping and antibody matched pair selection

Antibody matched pair selection was performed using a set of HRP conjugated and unconjugated mAb in sandwich ELISA in the presence of recombinant (S) protein. The HRP-conjugation of one mAb (SpMA-01) was performed using EZ-link Plus activated peroxidase labeling kit (ThermoFisher Scientific, USA) and titrated in an indirect ELISA prior to use in epitope mapping (data not shown). Briefly, the 96-well microplate was coated with 50 µL of 2 µg/mL of unconjugated/capture antibodies (SpMA-02) in coating buffer (pH-9.6) and incubated overnight at 4°C. In the control wells, 50 µL purified normal mouse IgG (2 µg/mL) and 50 mM PBS were added. Next day the plate was washed with 50 mM PBS, and the non-specific sites were blocked with 1% milk protein in 50 mM PBS and incubated for 1hr at RT. After washing the plate, 50 µL of 1 µg/mL of (S) in 50 mM PBS was added to the wells and incubated for 1 hour at room temperature. The plate was washed with PBS-Tween-20, and the wells were incubated with 50 µL of optimum concentration of HRP-labeled detection antibody (SpMA-01) and incubated for 1 hour at room temperature. After washing the plates three times with PBS-T-20 (VWR, USA), 50 μL of TMB substrate was added to each well and incubated for 10 to 15 minutes. The plate was read at 450 nm in a Spectramax M-2 plate reader (Molecular Devices, USA) after adding 50 μL of stop solution (5 N sulfuric acid) to each well.

### Synthesis of gold nanoparticles

All glassware used in the synthesis of gold nanosphere particles was washed thoroughly with deionized water followed by ultrapure water before use. Citrate-capped spherical gold nanoparticles (AuNPs) were synthesized following a method previously described (27). Briefly, 50 mL of 0.02% gold (III) chloride solution (Sigma, USA) was boiled in distilled water and 1.2 mL of 1% sodium citrate solution (Merck, USA) was added immediately with constant stirring. A color change from grey to blue and purple-violet occurred within 50 - 60 seconds. After the color change, the heat was turned off, the solution was stirred for 2 - 3 minutes, and allowed to cool at room temperature. The final colloidal solution suspension was characterized by ultraviolet-visible spectroscopy (UV-Vis) by scanning between 400 and 800 nm. The batch having λ-max between 525 and 535 nm was used to prepare antibody conjugate. Enhanced colloidal stability was measured using NaCl solution.The gold particles were stable in normal water solution but started aggregating in different concentration of NaCl (125mM, 250mM, 500mM and 1M). (**Supplementary Material Figure S1**).

### Conjugation of gold nanoparticles with monoclonal antibody

The anti-spike mAb (SpMA-01) of IgG1 isotype was selected for conjugation with a colloidal gold nanoparticle. The conjugation was optimized as described (28). Briefly, 6 to 10 µL of 1% K_2_CO_3_ (to adjust the pH to 6.5) was added to 1ml of colloidal gold solution in a glass tube, followed by 5 to 6 µg of anti-S SpMA-01 mAb and vortexed. The mAb-colloidal gold solution was incubated for 2 to 5 minutes at room temperature, followed by the addition of 20% BSA (final concentration approximately 0.1%), and the contents were transferred to an Eppendorf tube and centrifuged for 5 minutes in an Eppendorf centrifuge at 5000 RPM. The supernatant was removed, the pellet dissolved with 50 µL gold conjugation buffer.

### Assembly of lateral flow immunoassay strips

The LFIA strips were assembled using nitrocellulose (NC) membrane fixed on 10 mils polystyrene backing with high protein binding capacity (FF80HP plus LAM, Cytiva, USA), with pore sizes of 12 and 15 µm; and wicking time of 60 to 100 seconds. A single-layer matrix (Fusion-5, Cytiva, USA) was used as the sample and conjugate pad without any specific pre-treatment for uniform movement of gold nanoparticle conjugates. The absorbent pad (CF 7 Cytiva, USA), with high absorbance capacity, 100% cotton linter material (1873 µm thickness at 53kPA, Cytiva, USA) was overlapped at the top on the NC membrane.

### Performance of LFIA strips and spike protein titration

The performances of the strips composed of the NC membrane, the single-layer conjugate pad, and the absorbent pad were tested. The strips were manually cut into 6 × 0.5 cm and housed in a plastic cassette. The test and control lines were spotted manually in a circle by spotting 2 µg/dot of anti-spike mAb (SpMA-02) and 1 µg/dot of goat anti-mouse antibody (I-0759, Sigma, USA) at the reading window to give the test (T) and control line (C), respectively. To 10 µL of colloidal gold conjugated anti-spike mAb (SpMA-01), an equal volume of 10% alkali-treated casein was mixed and placed onto a conjugate pad. The membranes were dried for 5 to 7 minutes at room temperature to immobilize antibodies. Different concentrations of recombinant S ranging from 200 ng to 12.5 ng in 150 µL buffer and the control Vero cell extract [200 ng in 150 µL of PBS (50 mM PBS, pH 7.4)] were placed onto the sample application point. Driven by capillary forces, the immunocomplex migrated up the membrane into the absorbent pad and the test results were evaluated visually after 10 -15 minutes, the test results were evaluated visually. The selection of the optimal concentrations of the spike protein was visually inspected for T and C line results. The limit of detection (LOD) was calculated accordingly.

### LFIA running protocol

Purified anti-spike protein monoclonal antibodies (SpMA-01 and SpMA-02) were diluted in 50 mM PBS buffer (pH 7.4), 2 µg of capture mAb (SpMA-02) and 1 µg of goat anti-mouse antibody (Bethyl Lab, USA) and dropped onto a nitrocellulose membrane at the reading window to give the T and C, respectively. To 10 µL of colloidal gold conjugated anti-spike mAb (SpMA-01), an equal volume of 10% alkali-treated casein was mixed and placed onto a conjugate pad. The membranes were then dried at room temperature to immobilize antibodies. Samples of SARS-CoV-2 and its variants, or viruses and bacteria from the FDA pathogen panel were either diluted in PBS or treated with 100 mM TERGITOL-NP (prepared by mixing 334μL 100 mM Tergitol NP-9 with 666μL 100 mM Tergitol NP-10) followed by dilution with 150 µL of PBS. The samples were then placed onto sample application wells. Driven by capillary forces, the immunocomplex migrated up the membrane into the absorbent pad and after 10 to 15 minutes, the test results were evaluated visually. The selection of the optimal concentrations of the spike protein or pathogen antigens were visually inspected for Test (T-) and Control (C-) results. To determine the analytical sensitivity of spike protein in saliva or nasal samples, 200 ng of recombinant (S) was spiked into swabs and a 150μL extract was tested in the assay.

## Results

### Expression of full-length spike protein in Expi293F cells

**Figure 2:**
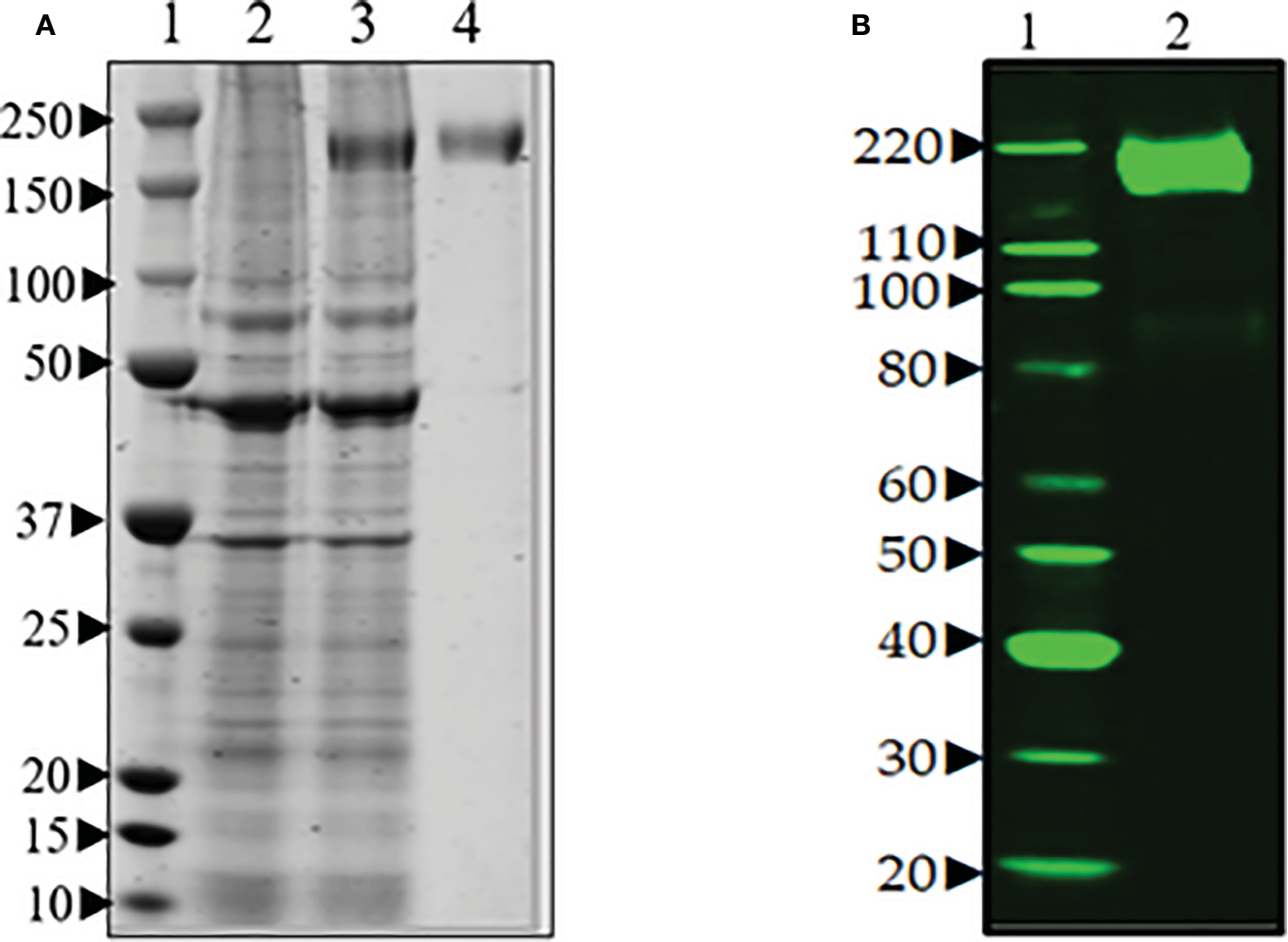
**(A)** SDS-PAGE of transient production of recombinant spike protein in Expi293F cells transfected with plasmid encoding spike gene under optimized conditions. Lane 1. protein marker, Lane 2. supernatant from untransfected cells, Lane 3. supernatant from transfected cells, and Lane 4. purified spike (~190 Da). **(B)** Western blot analysis of the purified spike protein with anti-hexa-His tag antibody confirmed the expression of spike protein by the Expi293F cells. Lane 1. Magic marker, Lane 2. purified spike protein.

The expression of spike protein was analyzed after transfection of Expi293F cells with the spike protein-encoding plasmid pCAGGS (BEI Resources NR-52394). The elevated expression level of a protein corresponding in size to the spike protein was visible upon aqua-staining of total proteins of spike cell lysates separated by SDS-PAGE when compared to a lysate of untransfected cells (**Figure 2A**). Western blot analysis with anti-hexa-His tag antibody confirmed the significant and specific expression of the spike protein as shown by a band of the expected size for the 190 kDa monomer in the transfectant (**Figure 2B**).

### Generation and characterization of anti-spike protein monoclonal antibodies

**Figure 3:**
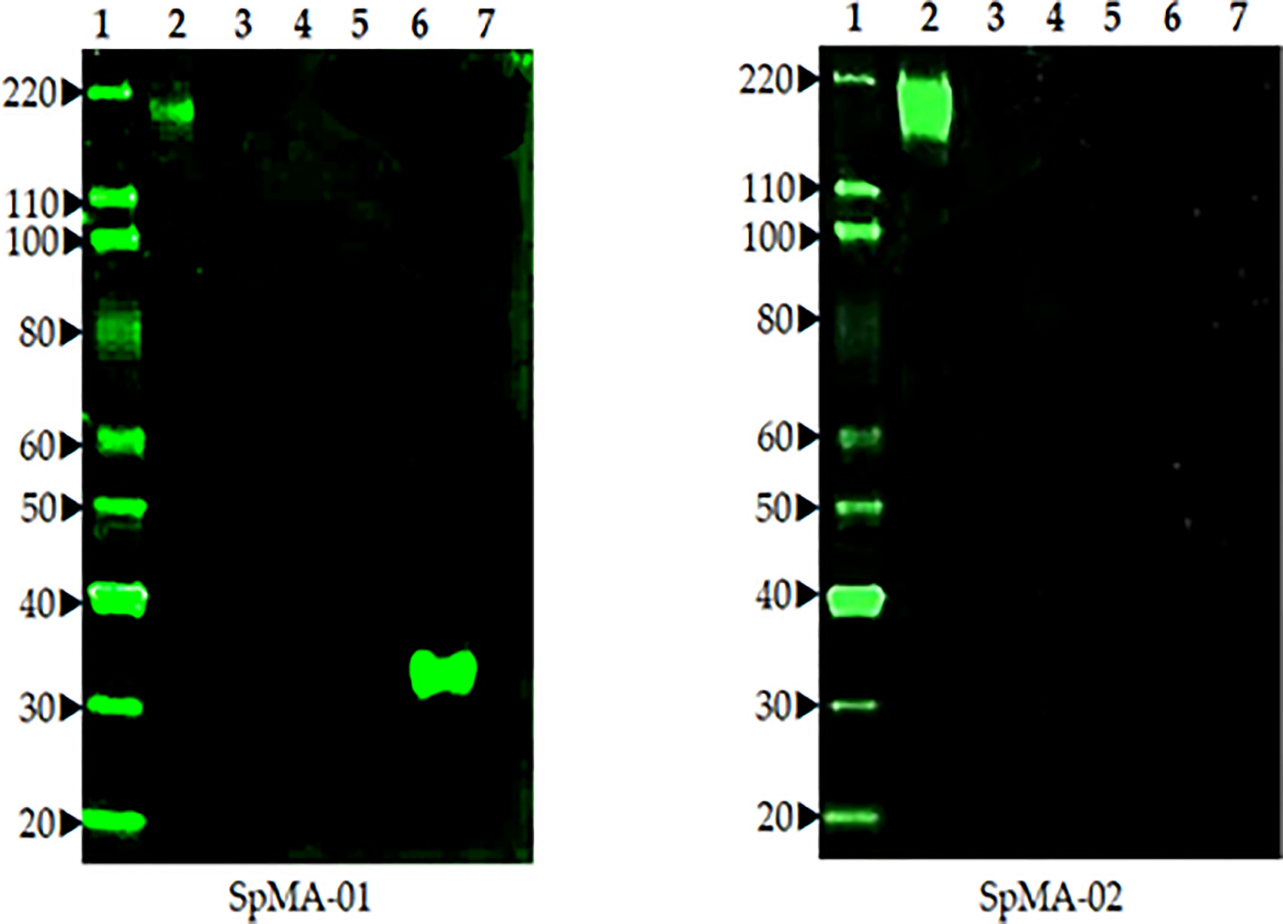
The Western blot analysis of two anti-spike mAbs (SpMA-01 and SpMA-02) with coronavirus proteins. Lane 1. Magic marker, Lane 2. NR-52397 SARS-CoV-2 full-length recombinant Spike protein, Lane 3. NR 53713 Spike protein human Coronavirus HKU1, Lane 4. NR-53591 MERS coronavirus, Lane 5, NR-52286 SARS-Related CoV-2, Lane 6, NR-52366 SARS-CoV-2 RBD recombinant protein, Lane 7. NR-53258 Vero-Cell E6 lysate (negative control).

Fusion of spleen cells from spike protein immunized mice with Sp2/O myeloma cells enabled the generation of anti-spike antibody-producing hybridoma cell lines. To identify cell lines that produce IgG specific for S, hybridoma supernatants were screened by ELISA using full-length recombinant S. Supernatants from ELISA-positive wells were subsequently tested by Western blot (data not shown). A total of eight hybridoma cell lines that demonstrated high-level reactivity against the full-length recombinant (S) by ELISA were selected (data not shown). These cell lines were cloned by limiting dilution to obtain a single hybridoma clone producing mAbs. Two mAbs (SpMA-01 and SpMA-02) of IgG1(κ) isotype were selected for this study and analyzed for antigen specificity in western blot with SARS-CoV-2 full-length recombinant (S) (NR-52397), HCoV HKU1 (NR-53713), MERS Coronavirus (NR-53591), SARS-Related Coronavirus-2 (NR-52286), SARS-CoV-2 RBD recombinant protein (NR-52366), and Vero-Cell E6 Lysate as control (NR-53258) (**Figure 3**). The mAbs were highly specific, and no cross-reactivity was observed. Moreover, the two mAbs recognized different epitopes on the recombinant S protein. SpMA-01 showed binding with both recombinant S and recombinant S RBD protein, which proved RBD binding epitopes on the S1 site of spike protein; however, mAb (SpMA-02) only recognized the epitope on (S).

### LFIA optimization and determination of limit of detection

**Figure 4:**
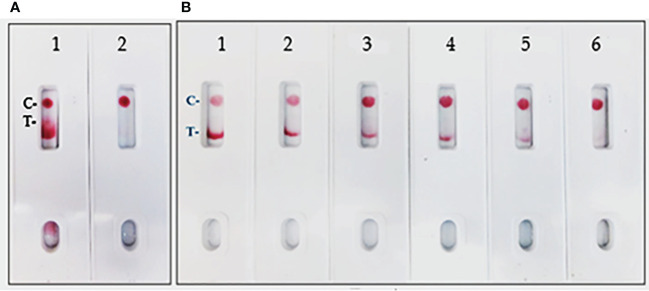
**(A)** LFIA Development with r-Spike protein. 1. NR-52397 SARS-CoV-2 r-Spike protein (200 ng), and 2. NR-53258 Vero E6 cell lysate (negative control). **(B)** Dose-response curve for LFIA using r-Spike protein. 1. NR-52397 SARS-CoV-2 r-Spike protein (200 ng), 2. 100 ng, 3. 50 ng, 4. 25 ng, 5. 12.5 ng and 6. NR-53258 Vero E6 cell lysate. The LOD for the Spike protein was 25 ng/test in a sample of 150 µL.

In the current assay, the recombinant S protein forms a bridge between gold nanoparticle conjugated mAb (SpMA-02) on one binding site of spike protein and unconjugated mAb (SpMA-01). The assay was optimized by employing SARS-CoV-2 recombinant S (200 ng/150 µL buffer), and Vero E6 cell extract (200 ng/150 µL buffer) as control. The SpMA-01 mAb allows the immunocomplex to be captured by spotting on the test line of the strip, forming a gold nanoparticle-conjugated bridging complex and producing a purple-colored T, while the goat anti-mouse antibody captures the unbound colloidal gold nanoparticle-conjugated SpMA-02mAb producing a purple-colored (C-). Control experiments of the strips with Vero cell E6 extract in buffer alone produced a purple band in the control (C-) area on the device but no band in the test (T-) area (**Figure 4A**). The LOD was determined by titrating the r-spike protein ranging from 200 ng - 12.5 ng in 150 µL buffer and the clear, purple-colored test band was achieved at a minimum concentration of 12.5 ng. According to the results, LOD was found to be 25 ng/test in 150 µL of PBS (**Figure 4B**). Additional optimization steps included the determination of the optimal antigen extraction reagent, buffer, and pre-incubation times. The most suitable results were obtained with 100 mM Tergitol surfactant used in antigen extraction for whole viruses and bacteria, 50 mM PBS (pH 7.4), and a 2-minute pre-incubation step. Optimized parameters selected for subsequent experiments are described below.

### LFIA specificity to spike/RBD proteins of SARS-CoV-2 and whole viruses

**Figure 5:**
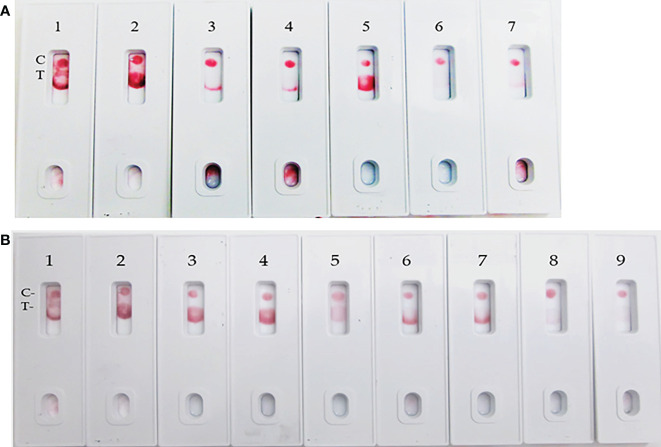
LFIA specificity with Coronavirus recombinant protein panel. **(A)** 1. NR-52397 SARS-CoV-2 spike protein (positive control), 2. NR-52366 SARS-CoV-2 RBD protein (positive control), 3. NR-52307 Spike RBD, Wuhan-Hu-1, Sf-9 insect cell produced-contains 223 residues of spike RBD, 4. NR-52946 Spike RBD from SARS-Related CoV-2 (HEK cell), 5. NR-52397 Full-length Spike protein-Wuhan-Hu-1 (stabilized), 6. NR-53713 Spike glycoprotein (stabilized), HKU1, 7. NR-53258 Vero E6 cell lysate (negative control). **(B)** 1. NR-52397 Spike protein (Stabilized) Coronavirus 2, (Wuhan-Hu-1) (positive control), 2. NR-52366 SARS-CoV-2 RBD protein (positive control), 3. NR-52397 Spike protein (Stabilized) Coronavirus 2, (Wuhan-Hu-1), 4. NR-53800 Spike Glycoprotein S2 Extracellular Domain (ECD) from SARS-CoV-2, 5. NR-53798 Spike Glycoprotein S1 Domain from SARS-CoV-2, 6. NR-52724 Spike protein from SARS-CoV-2 (S-2Pdfv), 7. NR-52946 Spike RBD from SARS-Related CoV-2 (HEK cell), 8. NR-53590 Spike Glycoprotein (Stabilized) from SARS Coronavirus, Tor-2 with C-Terminal, 9. NR-53258 Vero E6 cell lysate (negative control).

The LFIA was specific to various coronavirus recombinant proteins reported in (**Table 1**). The LFIA recognized all the SARS-CoV-2 spike Glycoprotein/RBD proteins very well, except for the truncated RBD proteins of SARS-CoV-2 Wuhan-Hu-1, which lacks signal sequences and contains 223 residues with a C-terminal His-tag (BEI Resources NR-52307), and the Wuhan-Hu-1 spike RBD protein fused to an N-terminal signal sequence and contains residues 319 to 541 with a C-terminal His-tag (NR-52946). The S glycoprotein of human coronavirus HCoV HKU1 (NR-53713) was also not recognized (**Figure 5A**). However, as shown in **Figure 5B**, the assay was also specific to Spike Glycoprotein S2 Extracellular Domain (ECD) from SARS-Coronavirus 2 (NR-53800), Spike Glycoprotein S1 Domain from SARS-Coronavirus-2 (NR-53798), Spike Glycoprotein (Stabilized) from SARS-Coronavirus 2 (NR-52724), and Spike RBD from SARS-Related Coronavirus 2 (NR-52946), (Wuhan-Hu-1), but did not recognize Spike Glycoprotein (Stabilized) from SARS Coronavirus, Tor-2 with C-terminal Histidine and Strep^®^ II Tags (NR-53590).

**Table 1:** LFIA Specificity with full-length Spike/RBD proteins of SARS-CoV-2 and whole viruses.

BEI No.	Spike proteins of SARS-CoV-2 and whole viruses www.beiresources.org	LFIA Results	Figure No.
**NR-52397**	Full-length (Stabilized) SARS-CoV-2 spike protein	+++	(**Figure 5A**)
**NR-52366**	SARS-CoV-2 RBD protein Wuhan-Hu-1	+++	(**Figure 5A**)
**NR-52307**	Spike RBD, Wuhan-Hu-1, contains 223 residues of spike RBD.	++	(**Figure 5A**)
**NR-52946**	Spike RBD from SARS-Related Coronavirus 2 (HEK cell),	++	(**Figure 5A**)
**NR-52397**	Full-length (Stabilized) SARS-CoV-2 spike protein	+++	(**Figure 5A**)
**NR-53713**	Spike glycoprotein (stabilized), HKU1	Negative	(**Figure 5A**)
**NR-53800**	Spike glycoprotein S2 Extracellular Domain (ECD) from SARS-coronavirus 2	+++	(**Figure 5B**)
**NR-53798**	Spike glycoprotein S1 domain from SARS-Coronavirus-2	+++	(**Figure 5B**)
**NR-52724**	Spike protein from SARS-Coronavirus 2 (S-2Pdfv)	+++	(**Figure 5B**)
**NR-52946**	Spike RBD from SARS-Related Coronavirus 2 (HEK cell),	+++	(**Figure 5B**)
**NR-53590**	Spike glycoprotein (stabilized) from SARS coronavirus, Tor-2 with C-terminal histidine and *Strep* ^®^ II tags	Negative	(**Figure 5B**)
**NR-52286**	Heat inactivated SARS-Related Coronavirus 2, Isolate USA-WA1/2020	Negative	(**Figure 6**)
**NR-52289**	SARS-Related Coronavirus 2, Isolate USA-WA1/2020, Gamma-Irradiated	Negative	(**Figure 6**)
**NR-52725**	Human Coronavirus OC43	Negative	(**Figure 6**)
**NR-52726**	Human Coronavirus 229E	Negative	(**Figure 6**)
**NR-470**	Human coronavirus NL63	Negative	(**Figure 6**)
**NR-53591**	Human coronavirus (MERS-CoV), EMC/2012	Negative	(**Figure 6**)

++, moderate positive; +++, strong positive

### Assay specificity to human coronaviruses

**Figure 6:**
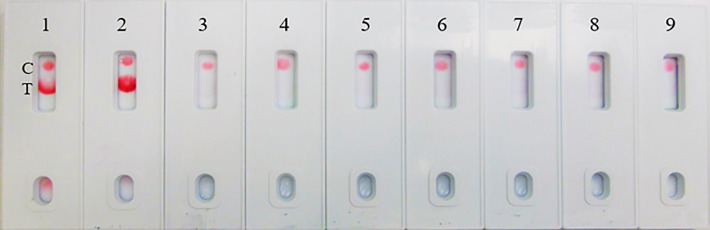
LFIA Specificity/Cross-reactivity with Corona Virus Panel. 1. NR-52397 SARS-CoV-2 spike protein (positive control), 2. NR-52366 SARS-CoV-2 RBD protein (positive control), 3. NR-52286 Heat inactivated SARS-Related Coronavirus 2, Isolate USA-WA1/2020, 4. NR-52289 SARS-Related Coronavirus 2, Isolate USA-WA1/2020, Gamma-Irradiated, 5. NR-52725 Human Coronavirus OC43, 6. NR-52726 Human Coronavirus 229E, 7. NR-470 Human coronavirus NL63, 8. NR-53591 Human coronavirus (MERS-CoV), EMC/2012, 9. NR-53258 Vero Cell E6 Lysate (negative control).

The assay specificity was verified with various human coronaviruses (**Table 1**). The LFIA did not show positive results with whole heat inactivated (NR-52286) and gamma irradiated (NR-52289) SARS-Related Coronavirus 2, Isolate USA-WA1/2020, and Human coronavirus OC43 (NR-52725), Human coronavirus 229E (NR-52726), Human coronavirus NL63 (NR-470) and MERS-CoV, EMC/2012.) (NR-53591) (**Figure 6**).

### Assay specificity to spike proteins of SARS-CoV-2 variant viruses

**Figure 7:**
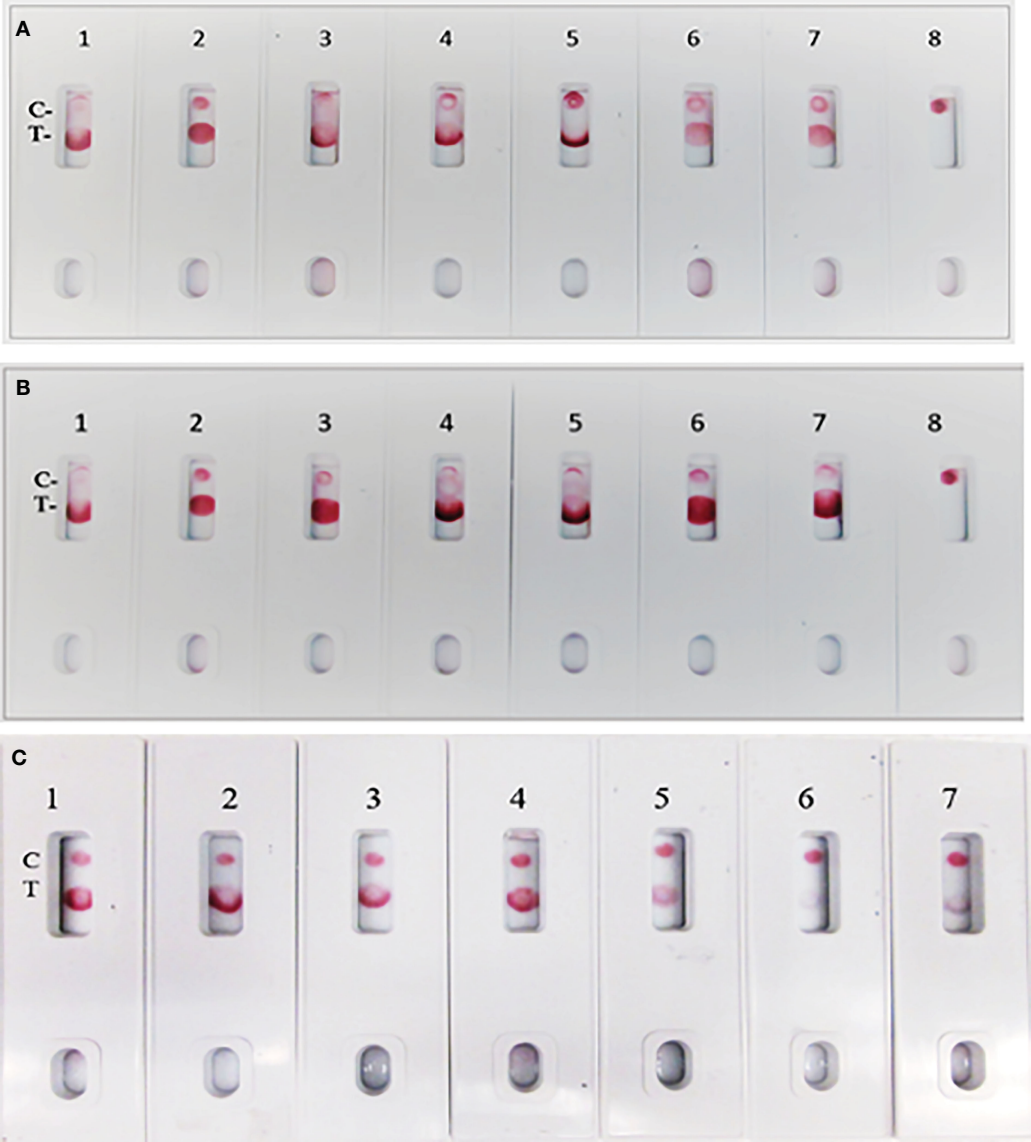
Assay specificity with spike protein of SARS-CoV-2 variant virus proteins. **(A)** 1. NR-52397 SARS-CoV-2 spike protein (positive control), 2. NR-52366 SARS-CoV-2 RBD protein (positive control), 3. NR-55614 Full-length SARS-Cov-2 Spike protein. B.1.617.2, 4. NR-55615 Full-length SARS-Cov-2 Spike protein. B.1.1.1, 5. NR-55616 Full-length SARS-Cov-2 Spike protein. A.23.1, 6. NR-53798 SARS-CoV-2 (COVID-19) S1 protein (N501Y), 7. NR-55420 SARS-CoV-2 (COVID-19) S1 protein (P681H), 8. NR-53258 Vero Cell E6 Lysate (negative control). **(B)** 1. NR-52397 SARS-CoV-2 spike protein (positive control), 2. NR-52366 SARS-CoV-2 RBD protein (positive control), 3. NR-55633 Spike Glycoprotein (Stabilized) from SARS-Related Coronavirus (P3), 4. NR-55614 Full-length SARS-Cov-2 Spike protein. B.1.617.2, 5. NR-55632 Full-length SARS-Cov-2 Spike protein. R.1, 6. NR-55277 Recombinant spike protein RBD, SARS-CoV-2 (UK variant), 7. NR-55278 Recombinant spike protein RBD, SARS-CoV-2 (South African variant), 8. NR-53258 Vero Cell E6 Lysate (negative control). **(C)** 1. NR-52397 Spike SARS-CoV-2 stabilized protein (positive control), 2. NR-52366 SARS-CoV-2 RBD Protein (positive control), 3. NR-55307 stabilized spike trimer SARS-CoV-2 (P.1), 4. NR-55310 stabilized spike trimer SARS-CoV-2 (B.1.351), 5. NR-55311 stabilized spike trimerSARS-CoV-2 (B.1.1.7), 6. NR-55438 stabilized spike trimerSARS-CoV-2 (B.1.526), 7. NR-53258 Vero Cell E6 Lysate (negative control).

During the pandemic, several SARS-CoV-2 variants evolved with multiple overlapping mutations to the spike protein and a portion of the spike protein RBD. We evaluated the specificity of the LFIA using spike proteins of several Variant viruses (**Table 2**) below. The LFIA recognized all the spike proteins of the variants except the stabilized spike trimer of B.1.526. The results in **Figure 7A** show specific binding with the full-length SARS-CoV-2 Spike protein of B.1.617.2, B.1.1.1, A.23.1, SARS-CoV-2 S1 protein (N501Y), and S1 protein (P681H). The results in **Figure 7B** show specific binding with the Spike protein of Coronavirus (P3), B.1.617.2, R.1, and recombinant spike RBD proteins of the UK and South African variants. Also, the results in **Figure 7C** show specific binding with the stabilized spike trimer SARS-CoV-2 B.1.351, P.1, B.1.1.7, but not with stabilized spike trimer of SARS-CoV-2 B.1.526.

**Table 2:** LFIA Specificity with full-length Spike/RBD proteins of SARS-CoV-2 variants

BEI No.	Spike/RBD proteins of SARS-CoV-2 Variants www.beiresources.org	LFIA Results	Figure No.
**NR-55614**	Full-length SARS-Cov-2 Spike protein. B.1.617.2.	+++	(**Figure 7A**)
**NR-55615**	Full-length SARS-Cov-2 Spike protein. B.1.1.1	+++	(**Figure 7A**)
**NR-55616**	Full-length SARS-Cov-2 Spike protein. A.23.1.	+++	(**Figure 7A**)
**NR-53798**	SARS-CoV-2 (COVID-19) S1 protein (N501Y)	+++	(**Figure 7A**)
**NR-55420**	SARS-CoV-2 (COVID-19) S1 protein (P681H)	+++	(**Figure 7A**)
**NR-55633**	Spike Glycoprotein (Stabilized) from SARS-Related Coronavirus (P3)	+++	(**Figure 7B**)
**NR-55632**	Full-length SARS-Cov-2 Spike protein. R.1.	+++	(**Figure 7B**)
**NR-55277**	Recombinant spike protein RBD, SARS-CoV-2 (UK variant)	+++	(**Figure 7B**)
**NR-55278**	Recombinant spike protein RBD, SARS-CoV-2 (South African variant)	+++	(**Figure 7B**)
**NR-55307**	Stabilized spike trimer SARS-CoV-2 (P.1)	+++	(**Figure 7C**)
**NR-55310**	Stabilized spike trimer SARS-CoV-2 (B.1.351)	+++	(**Figure 7C**)
**NR-55311**	Stabilized spike trimer SARS-CoV-2 (B.1.1.7)	+++	(**Figure 7C**)
**NR-55438**	Stabilized spike trimerSARS-CoV-2 (B.1.526)	Negative	(**Figure 7C**)

+++, strong positive

### Assay specificity/cross-reactivity with FDA-approved pathogen Panel (respiratory virus, influenza virus, bacteria and fungi)

**Figure 8:**
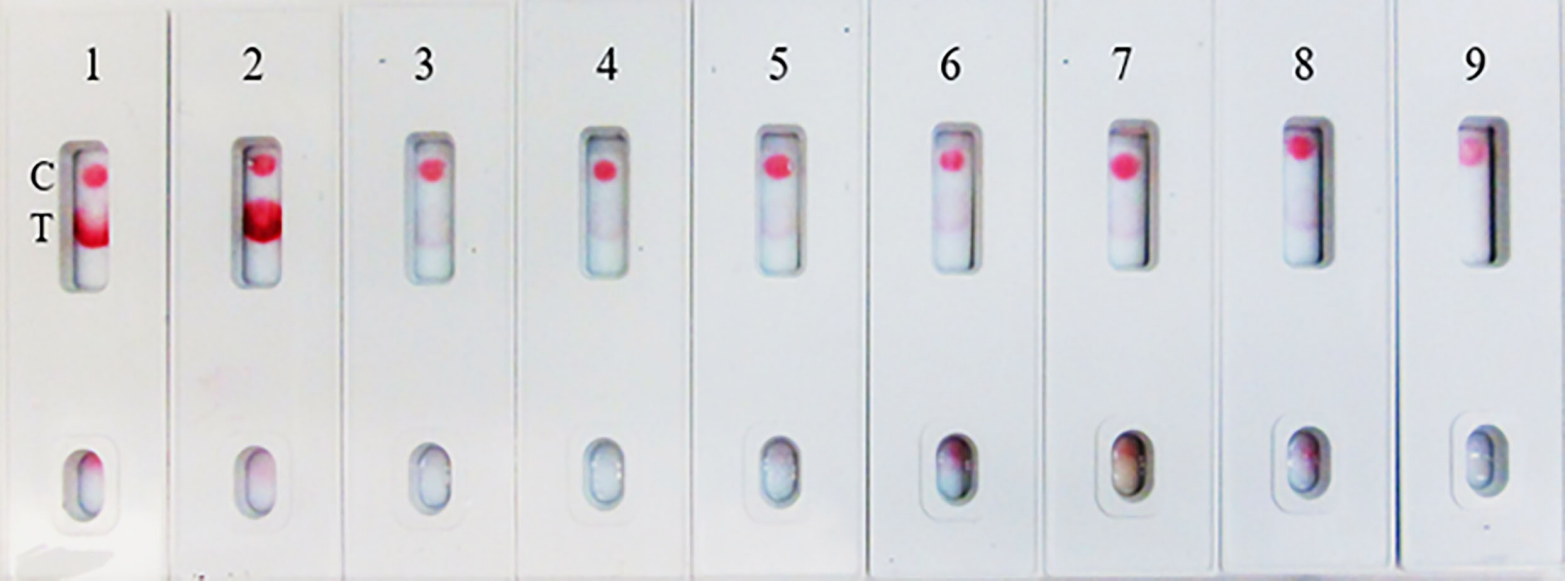
LFIA Specificity/Cross-reactivity with Respiratory Virus Panel. 1. NR-52397 SARS-CoV-2 spike protein (positive control), 2. NR-52366 SARS-CoV-2 RBD protein (positive control), 3. NR-28528 Human Respiratory Syncytial Virus A1998/12-21, 4. NR-4052 Respiratory Syncytial Virus B1, 5. NR-12149 Respiratory Syncytial Virus A2, 6. NR-51452 Rhinovirus Type-35 (164A), 7. NR-51453 Rhinovirus Type-40 (1794), 8. NR-22234 Human Metapneumovirus, 9. NR-53258 Vero Cell E6 Lysate (negative control).

**Figure 9:**
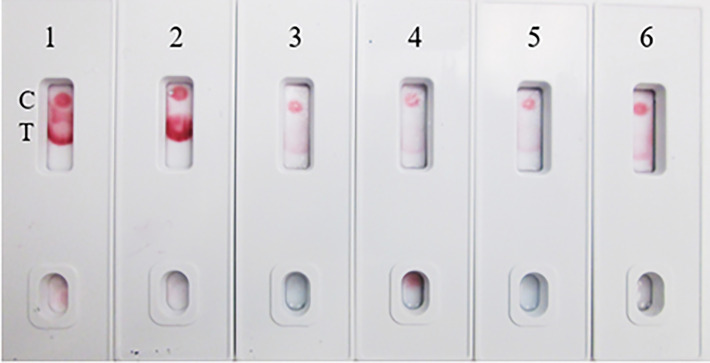
LFIA Specificity/Cross-reactivity with Influenza Virus Panel. 1. NR-52397 SARS-CoV-2 spike protein (positive control), 2. NR-52366 SARS-CoV-2 RBD protein (positive control), 3. NR-42006 Influenza B Virus, B/Brisbane/33/2008 (Victoria Lineage), 4. NR-42007 Influenza A Virus, A/Wisconsin/15/2009 (H3N2), 5. NR-48680 Human parainfluenza virus, 1 HPIV1/FRA/29221106/2009, 6. NR-53258 Vero Cell E6 Lysate (negative control).

**Figure 10:**
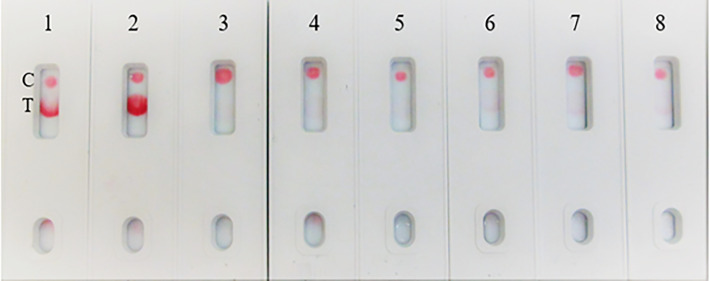
LFIA Cross-reactivity with Bacteria and Fungus Panel. 1. NR-52397 SARS-CoV-2 spike protein (positive control), 2. NR-52366 SARS-CoV-2 RBD protein (positive control), 3. NR-51849 Streptococcus pneumoniae SPEC1, 4. NR-46376 Staphylococcus epidermidis VCU013, 5. NR-51329 Pseudomonas aeruginosa EnvKY1, 6. NR-42457 Bordetella pertussis H921, 7. NR-29340 Candida albicans 23B, 8. Mucin (Sigma) (negative control).

The assay specificity was verified with a respiratory pathogen Panel (**Table 3**). No cross-reactivity was observed to human respiratory syncytial viruses, rhinoviruses and human metapneumorivus virus (**Figure 8**). Human influenza and parainfluenza viruses (**Figure 9**) as well as *Streptococcus*, *Pseudomonas, Bordetella pertussis* and *Candida albicans* (**Figure 10**) all showed negative results.

**Table 3:** LFIA Specificity/Cross-reactivity with FDA approved pathogen Panel.

BEI No.	Virus and Bacterial Pathogens/strains www.beiresources.org	Cross-reactivity	Figure No.
**NR-22234**	Human metapneumovirus TN/91-320	Negative	(**Figure 8**)
**NR-4052**	Human respiratory syncytial virus B1	Negative	(**Figure 8**)
**NR-12149**	Human respiratory syncytial virus A2	Negative	(**Figure 8**)
**NR-51452**	Rhinovirus 35 164A	Negative	(**Figure 8**)
**NR-51453**	Rhinovirus 40 1794	Negative	(**Figure 8**)
**NR-28528**	Human respiratory syncytial virus A1998/12-21	Negative	(**Figure 8**)
**NR-48680**	Human parainfluenza virus 1 HPIV1/FRA/29221106/2009	Negative	(**Figure 9**)
**NR-42007**	Influenza A Virus, A/Wisconsin/15/2009 (H3N2	Negative	(**Figure 9**)
**NR-42006**	Influenza B Virus, B/Brisbane/33/2008 (Victoria Lineage)	Negative	(**Figure 9**)
**NR-51849**	*Streptococcus pneumoniae* SPEC1	Negative	(**Figure 10**)
**NR-46376**	*Staphylococcus epidermidis* VCU013	Negative	(**Figure 10**)
**NR-51329**	*Pseudomonas aeruginosa* EnvKY1	Negative	(**Figure 10**)
**NR-42457**	*Bordetella pertussis* H921	Negative	(**Figure 10**)
**NR-29340**	*Candida albicans* 23B Fungi	Negative	(**Figure 10**)

### Spike-and-recovery experiment

**Figure 11:**
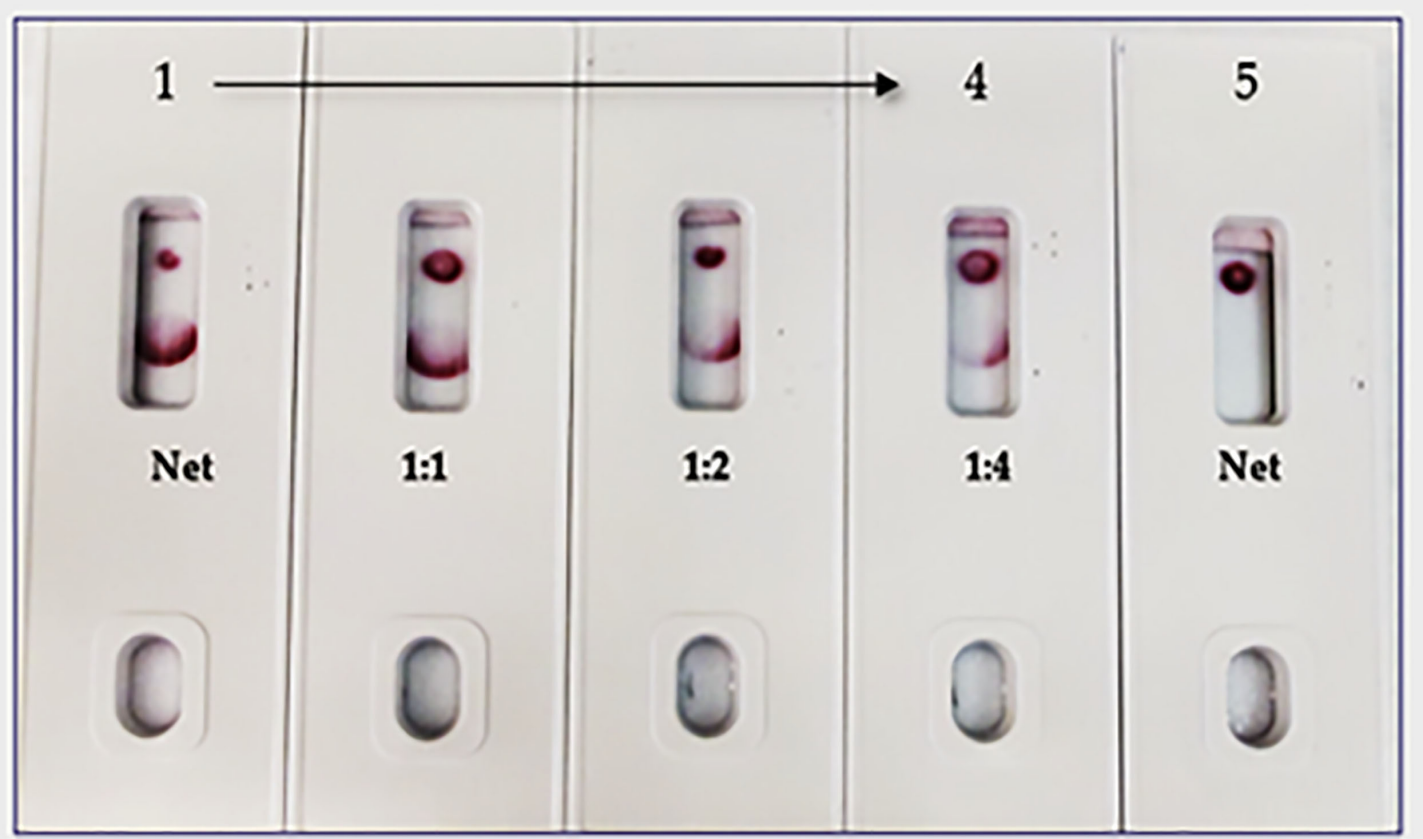
Throat swab, spiked with Covid-19 r-spike protein (200 ng), extracted and diluted, 2. (1:1 dilution), 3. (1:2 dilution), 4. (1:4 dilution), 5. Throat swab, spiked with Vero E6 Cell Lysate (200 ng) negative control. The assay could detect 25 ng of spike antigen in sample.

In the spike-and-recovery test, recombinant spike protein was either neat (200 ng/150 µL) or diluted (1:1, 1:2, and 1:4), spiked into cotton swabs and tested as dummy clinical specimens (**Figure 11**). The assay could detect less than 25 ng of spike antigen.

### Swab mimicking experiment

**Figure 12:**
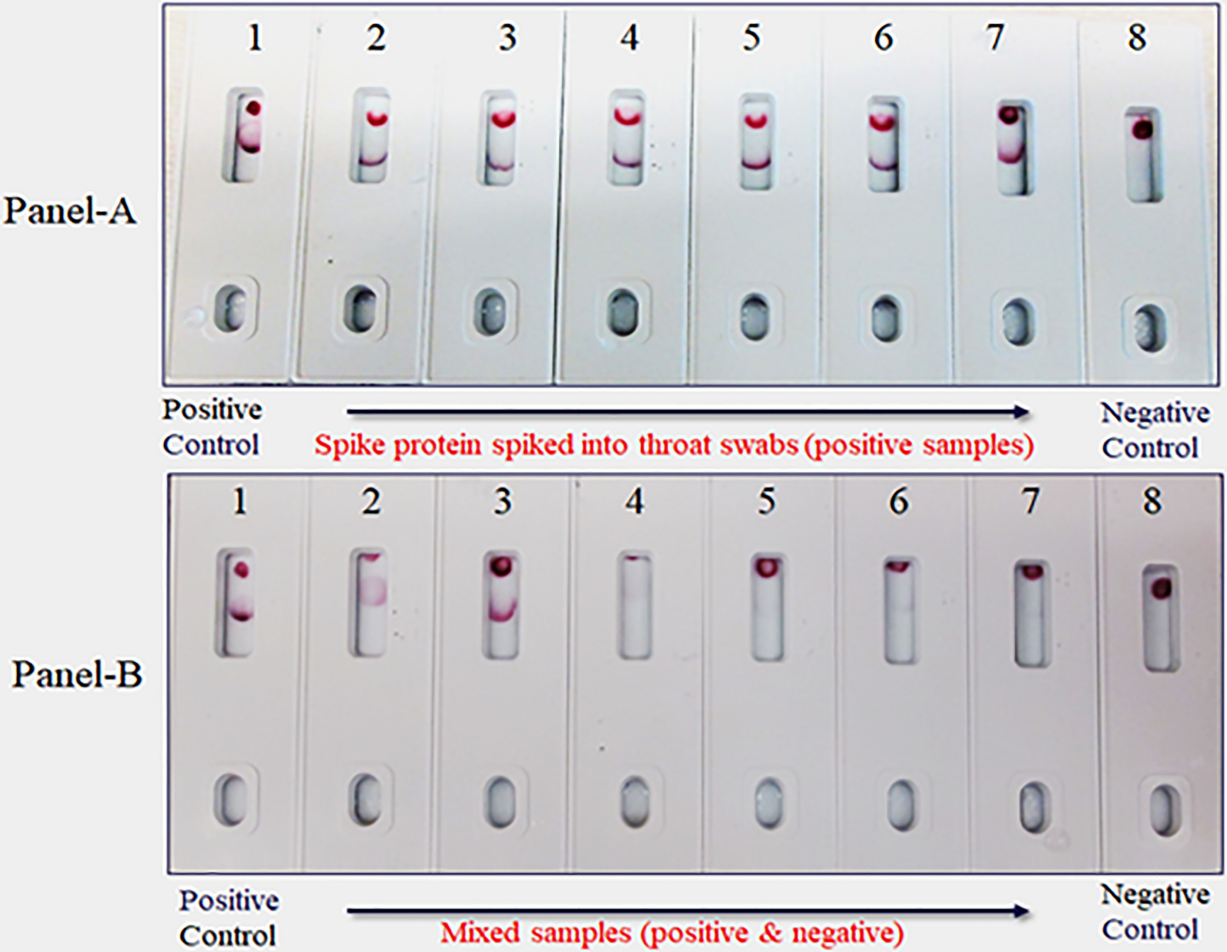
Covid-19 recombinant Spike protein spiked into Vero cell lysate and absorbed with swabs to mimic clinical samples and tested. The “Swab Mimicking Test” experiment was designed to test simultaneously spiking recombinant spike protein into Vero E6 cell lysate, absorbed, and extracted for test. Panel A: all positive samples, and Panel B: mixed both positive (1 to 3) and negative samples (4 to 8).

Due to unavailability of SARS-CoV-2 clinical specimens, the “Swab Mimicking Test” was performed using blinded samples (recombinant spike protein randomly spiked into saliva swabs), extracted the antigen, and tested. The assay could distinguish between positive and negative samples clearly and the positive concentration was within 25 ng range (**Figure 12**, Panel-A: all positive samples, and Panel B: mixed both positive and negative samples).

## Discussion

The majority of the SARS-CoV-2 point-of-care diagnostics are focused on the use of LFIA for antibody (IgG/IgM) or SARS-CoV-2’s Nucleocapsid protein (NP) detection. The FDA alone approved commercial antigen kits (12 out of 13) targeting the Nucleocapsid (NP). The alert issued by the CDC raised concerns regarding the devices’ tendency to exhibit false positive results (29). A similar warning was issued by the FDA (30). While this might be an inherent limitation of antibody and NP-based tests, it could possibly be mitigated by targeting a different antigen. One such potential target is the outer membrane full-length spike protein (S), which is exposed, thereby easy to detect without any extraction method. Herein, we describe the development of a novel alternative full-length spike antigen-based “Lateral Flow Immunoassay” (LFIA), comprising two high-affinity, and very specific monoclonal antibodies, directed against different epitopes on the full-length spike protein. The LFIA qualitatively detects the presence of the (S) protein in samples. The test reagent (150 µL of swab extract) is added to the well of the test card, and within 5-10 min, the result is displayed on a paper strip visualized through a small window on the front of the test card. To develop this assay, we generated a panel of mouse mAbs against purified recombinant full-length (stabilized) spike protein (190 kDa), expressed in Expi293F cells. Prior to the immunization of mice, the integrity of the purified (S) was assessed on denaturing SDS-PAGE and Western blot (WB) analysis (**Figures 1A, B**). The mAbs were screened by ELISA and characterized in WB and isotyping. The mAbs that exhibited high-affinity binding to (S) in ELISA were identified and empirically tested in a competitive sandwich ELISA for matched antibody pair selection. Two mAbs SpMA-01and SpMA-02 selected from functional assays as capture and detection antibodies for this assay. The antibody pairs further characterized in WB to find out the selective binding specificity with NR-52397-SARS-CoV-2 full-length recombinant (S), NR-52366-recombinant spike’s S1 protein and other known human pathogenic coronavirus antigens such as, NR-53713-Heat inactivated Human Coronavirus (HKU1), NR-53591-MERS Coronavirus, and NR-52286-SARS-Related Coronavirus-2. The WB revealed, the SpMA-01 recognizes epitope on the spike’s S1 domain (35 kDa), and SpMA-02 recognizes epitope on (S) respectively and no cross-reactive was observed with the human coronavirus antigens (**Figure 2**). Our observations suggest that SpMA-02 recognizes unique conformational epitopes on the full-length Spike (S) protein, which represent multiple points of contact between the S1 domain and beyond S1 domain (either N- or C- terminus). These points of contact may involve amino acids that are not contiguous in the antigen sequence, but rather come together in the folded S protein. These antibodies were also characterized for high specificity, and sensitivity for the detection of SARS-CoV-2 (S) protein in WB in the presence of other variants/mutants (data not shown). In the present study, we used colloidal gold as a reporter (31). The non-covalent adsorption of antibodies to gold particles is based on ionic interaction between the negatively charged nanoparticle and the positively charged sites on IgG. Preparing a high-quality colloid gold solution is a key step in ensuring the sensitivity of the test strip. We characterized the gold nanoparticles using Spectra-Max 5Me UV/Vis/NIR-spectrophotometer. The colloidal gold synthesized showed highest absorption at 525 nm. The absorbance at 525 nm was 2.41. The size of the gold nanoparticle was 50nm. Gold nanoparticles of varying sizes have different conjugation efficiencies under different pH values and antibody concentrations (32). We optimized the antibody conjugation process using 50 nm colloid gold nanoparticle solution (pH 6.5) and 6 µg of mAb/mL of gold solution. The evaluation of the “capture–detection probe pairs” allowed selection of the most suitable pair for the sensitive detection of SARS-CoV-2 antigen: capture probe, mAb, and detection probe, mAb. The LFIA was developed using capture–detection probe pairs and r-spike protein (200 ng/150µL/test) along with the same concentration of Vero cell lysate (negative control) (**Figure 4A**). To evaluate the sensitivity of the assay, we performed four different concentrations (i.e., 200, 100, 50, 25ng and 12.5 ng) of antigens (**Figure 4B**). The intensity of the test line is correlated with the (S) protein concentration, where samples containing lower amounts of (S) protein generated lighter test lines (T-). The goat anti-mouse antibody was used to prepare the strip’s control line (C-). The control line (C-) color development validates the gold conjugation of the mAb. Based on visual readout of the test results, this assay exhibits a limit of detection (LOD) of 25 ng/test. Under optimal conditions, the sensitivity of LFIA was determined by testing different dilutions of the full-length spike protein. As shown in **Figure 4B**, the Test (T-) line decreased with decreasing concentration of the specimens. When the concentration of the specimen was below 25 ng, the (T-) line reached the lowest value. Consequently, in our LFIA, the LOD is as low as 25 ng/150 µL. The reproducibility of the LFIA was verified with full-length spike proteins of coronaviruses including variants along with Vero E6 cell lysate as a negative control using the same batch of test strips (data not shown).

The assay exhibited 98% specificity with various spike proteins and with the pathogens. The LFIA specificity was analyzed with a coronavirus recombinant protein panel (**Table 1**), and detected very well the SARS-CoV-2 full-length spike and RBD proteins (as positive controls), but low LOD was observed with baculovirus-expressed spike RBD protein of Wuhan-Hu-1 containing 223 AA residues, the Spike RBD of SARS related coronavirus-2 (HEK Cells) of HKU1 and no reactivity was observed with Spike glycoprotein (stabilized) of HKU1 (**Figure 5A**). The logical inference is that the “capture-detection” antibody pairs recognize very specific epitopes which are not present on both the truncated spike RBD proteins, and also the Spike glycoprotein of HKU1. However, high level of binding specificity was observed with the SARS-CoV-2 Spike Glycoprotein (both S1 and S2 Extracellular Domains), Spike protein from SARS-CoV-2 (S-2Pdfv) and spike RBD from SARSCoV-2 (expressed in HEK cells). Conversely, the Spike glycoprotein (stabilized) from SARS Coronavirus-2 with Tor-2 C-Terminal sequence failed to detect; the underlying reason may be the antibody binding sites/epitopes of the “capture–detection” mAb pairs are not accessible in the presence of large Tor-2 protein sequence (**Figure 5B**). Also, the LFIA was very specific and no cross-reactivity was observed with heat-inactivated and gamma-Irradiated SARS-Related Coronavirus 2, Isolate USA-WA1/2020, Human Coronavirus OC43, Human Coronavirus 229E, Human coronavirus NL63 and Human coronavirus (MERS-CoV), EMC/2012 (**Figure 6**). Over the course of time numerous viral variants have been emerged which carry signature amino acid substitutions in key areas of the immunodominant spike protein, with evidence of altered virus characteristics (33). Therefore, we evaluated the specificity of the LFIA with several spike/RBD proteins from different variants (**Table 2**). The assay could detect the full-length spike proteins of (B.1.617.2, B.1.1.1, A.23.1, S1 protein (N501Y), S1 protein (P681H) (**Figure 7A**). The assay could detect the full-length spike proteins of (P3, R.1, SARS-CoV-2 UK and South African variants) (**Figure 7B**). The assay could also detect the full-length spike proteins of (P.1, B.1.351, B.1.1.7) (**Figure 7C**), but the assay did not detect the stabilized spike trimer of SARS-CoV-2 (B.1.526) Upon testing B.1.526 variant recombinant protein in ELISA with SpMA-01 and SpMA-02, we observed low affinity. This suggests that some of the amino acids of the putative S1 domain are hidden or else the protein does not fold properly during manufacturing. We did not evaluate the ability of this method to detect omicron variants, such as BA.4, BA.5, and BQ1.1. This would be our future plan as we move towards final development of this assay (34). Next, we evaluated the cross-reactivity and potential interference with the panel of FDA-approved pathogens, and a negative matrix (Mucin) (**Table 3**). No cross-reactivity was observed with Human Respiratory viruses (**Figure 8**), which include Human Respiratory Syncytial Virus A1998/12-21, Respiratory Syncytial Virus B1, Respiratory Syncytial Virus A2, Rhinovirus Type-35 (164A), Rhinovirus Type-40 (1794) and Human Meta-pneumo Virus. No cross-reactivity was observed with Influenza viruses (**Figure 9**), which include Influenza virus B Virus, B/Brisbane/33/2008 (Victoria Lineage), Influenza A Virus, A/Wisconsin/15/2009 (H3N2), and Human parainfluenza virus 1 HPIV1/FRA/29221106/2009. Also, no cross-reactivity was observed with several bacteria and fungi (**Figure 10**), including *Streptococcus pneumoniae* SPEC1, *Staphylococcus epidermidis* VCU013, *Pseudomonas aeruginosa* EnvKY1*, Bordetella pertussis* H921, and *Candida albicans* 23B.

The limitations of this study include the fact that we employed a modified method “spike-and-recovery” to perform a dummy clinical specimen test. Therefore, the recombinant SARS-CoV-2 full-length spike protein was spiked into throat swabs directly and extracted as a clinical specimen and added to device’s well (**Figure 11**). The throat swab, extracts were used either “Net”, or different dilutions (1:1 to1:4) along with Vero E6 cell Lysate (negative control). The assay could detect as low as 25 ng of spike antigen in the sample. We also designed the “Swab Mimicking Test” with the positive and negative samples by randomly spiking recombinant spike protein into saliva swabs to mimic clinical specimens. The assay was capable of distinguishing positive and negative samples. (**Figure 12**, Panel-A: all positive samples, and Panel B: mixed both positive and negative samples).

## Conclusion

To date, rapid diagnostics of COVID-19 to determine the antigen or the presence of IgG/IgM antibodies to SARS-CoV-2 to prevent the spread of the virus is carried out using lateral flow tests. SARS-CoV-2 antigen assays serve as rapid identification tests for in home use or for triage of patients who are likely to have COVID-19, reducing the need or wait time for confirmatory molecular tests. Monoclonal antibodies against the N protein of SARS-CoV-2 have been generated, and several rapid test kits are in the market (35), but of concern with these kits are common false positive and false negative results. In this work, we report the development and the detailed validation of a Lateral Flow Immunoassay (LFIA) platform based on gold nanoparticle conjugated anti-spike mAb for the detection of SARS-CoV-2 spike protein. Our LFIA detects the presence of viral full-length spike protein (S) through a conventional immunocapture format. The mAbs are highly specific for spike protein epitopes and showed no cross-reactivity with human coronaviruses (HCoV-OC43, HCoV-229E, HCoV-HKU1, and HCoV-NL63), however the assay could detect spike proteins of key SARS-CoV-2 variants (A.23.1, B.1.1.1, 1.617.2, B.1.1.7, B.1.351, P.1, R.1, N501Y, S1 protein of UK and South African). The assay is very specific and did not show cross-reactivity to an FDA-approved respiratory pathogens (RP) panel. The limitations of this study include the fact that we employed a modified method (spike recovery method) to test antigen spiked samples as dummy clinical specimen and the test results are remarkable good. Under the optimal assay conditions, only 150 μL of sample diluent is required to detect the SARS-CoV-2 antigen within 5-10 min. The assay is approximately 98% specific, and the LOD was as low as 25 ng/test of 150 µL for SARS-CoV-2 antigen. This LFIA is definitely suitable for point-of-care detection and provides a great application for SARS-CoV-2 epidemic control in third-world countries.

## Data availability statement

The original contributions presented in the study are included in the article/**Supplementary Material**. Further inquiries can be directed to the corresponding author.

## Ethics statement

Immunization of mice was performed in accordance with PHS Policy on Humane Care and Use of Laboratory Animals. Experiments were approved by the ATCC Institutional Animal Care and Use Committee (IACUC).

## Author contributions

SM designed the study and performed experiments. YW, JC, and CW contributed reagents, materials and developed tools. SM, RM, SR, and RB wrote the paper. All authors contributed to the article and approved the submitted version.
