# Supplementary material for: Development and validation of a rapid and easy-to-perform point-of-care lateral flow immunoassay (LFIA) for the detection of SARS-CoV-2 spike protein
Source: Front Immunol. 2023 Feb 23;14:1111644. doi: 10.3389/fimmu.2023.1111644 (PMC9995903; doi:10.3389/fimmu.2023.1111644)
Supplement: Supplementary file 1 [file DataSheet_1.pdf]

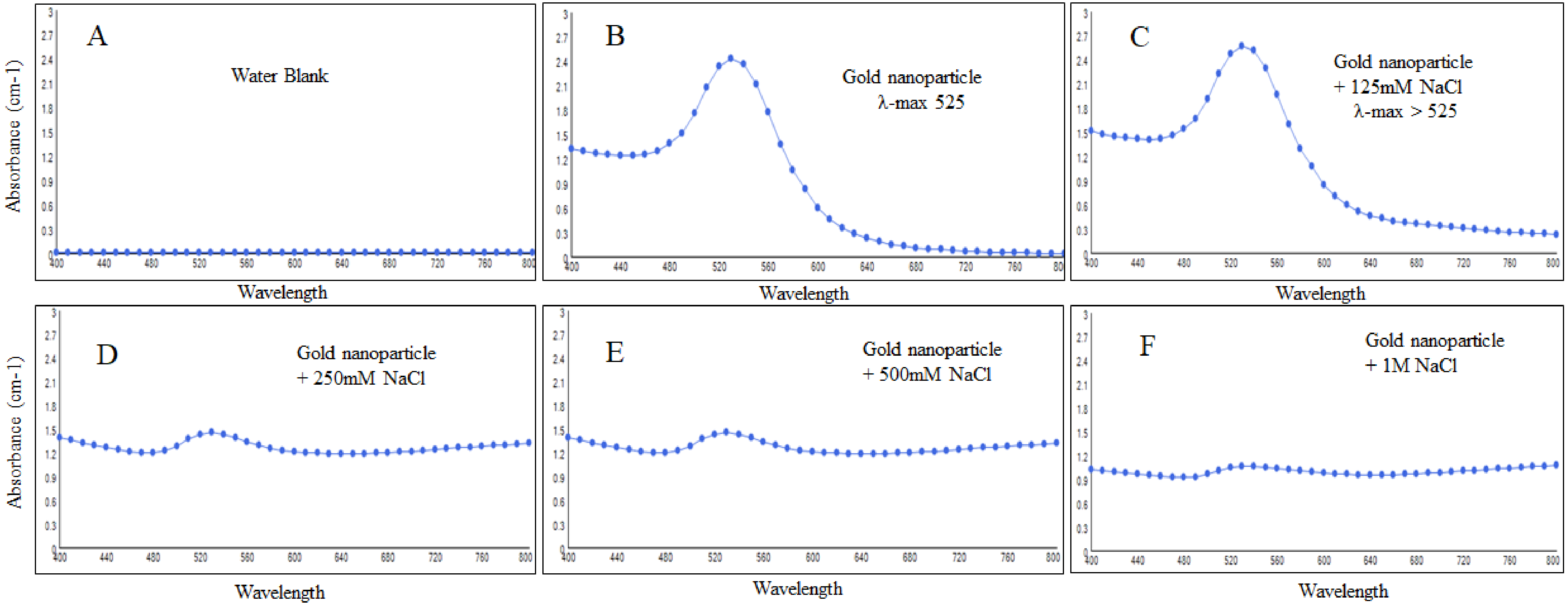

Supplementary Material, Fig S-1. Plasmon shift assays to analyze gold nanoparticle stability in presence of NaCl. The gold nanoparticles were analyzed in UV/VIS/NIR spectrophotometer for stability in presence NaCl, using scanning wavelength range (400-800nm). A-Water Blank. B-Normal gold nanoparticles in water (colloidal form λ-max 525). C-Gold nanoparticles, added 125mM NaCl (stability changed λ-max > 525). D-Gold nanoparticles, added 250mM NaCl (stability changed, start aggregating). E-Gold nanoparticles, added 500mM NaCl (stability changed, more aggregation). F-Gold nanoparticles, added 1M NaCl (stability changed, complete aggregation).
